# Phase Equilibria in the Nb-Rich Region of Al-Nb-Sn at 900 and 1200 °C

**DOI:** 10.3390/ma12172759

**Published:** 2019-08-28

**Authors:** Ioannis Papadimitriou, Claire Utton, Panos Tsakiropoulos

**Affiliations:** Department of Materials Science and Engineering, The University of Sheffield, Sir Robert Hadfield Building, Mappin Street, Sheffield S1 3JD, England, UK

**Keywords:** engineering and physical sciences research council (EPSRC)-rolls royce (RR) strategic partnership, phase equilibria, niobium, aluminium, tin, Nb_5_Sn_2_Al

## Abstract

The Al-Nb-Sn phase diagram was studied experimentally in the Nb-rich region to provide important phase equilibria information for alloy design of Nb-silicide based materials for aero engine applications. Three alloys were produced: Nb-17Al-17Sn, Nb-33Al-13Sn and Nb-16Al-20Sn (at.%). As-cast and heat-treated alloys (900 and 1200 °C) were analysed using XRD (X-ray diffraction) and SEM/EDS (scanning electron microscopy/ electron dispersive x-ray spectroscopy). Tin showed a high solubility in Nb_2_Al, reaching up to 21 at.% in the Sn-rich areas, substituting for Al atoms. Tin and Al also substituted for each other in the A15 phases (Nb_3_Al and Nb_3_Sn). Tin showed limited solubility in NbAl_3_, not exceeding 3.6 at.% as it substituted Al atoms. The solubility of Al in NbSn_2_ varied from 4.8 to 6.8 at.%. A ternary phase, Nb_5_Sn_2_Al with the tI32 W_5_Si_3_ crystal structure, was found to be stable. This phase was observed in the 900 °C heat-treated samples, but not in the 1200 °C heated samples.

## 1. Introduction

Nb-silicide based materials have been extensively studied as next generation alloys for aero engines due to their high melting temperatures, and excellent strength and creep resistance at high temperatures [1,2,3,4,5,6]. Alloy compositions can be complex in order to produce a balance of mechanical and oxidation properties at both high and ambient temperatures. To improve the oxidation resistance of the bulk material, Al and Sn may be added [7]. Tin is shown to improve oxidation resistance particularly in the pesting regime (temperature range 750–950 °C) [2,8,9,10,11]. Aluminium is also shown to improve both the oxidation resistance at higher temperatures whilst also lowering the density of the alloys [12]. However, oxidation resistance cannot be improved through alloying alone, and coatings will be required. Coating systems that form protective alumina and silica top-coats will play a pivotal role [13]. As such, the Al-Nb-Sn phase diagram is of interest and important for both the alloy design of these materials and possible coating systems. This work is part of a larger project to understand the phase equilibria in Nb-based alloys, and to improve oxidation resistance and mechanical properties of these materials through alloy design.

To date, the binary phase diagrams of the constituent elements have been thoroughly investigated. The Nb-Al and Nb-Sn systems consist of the NbAl_3_, Nb_2_Al and Nb_3_Al [14], and NbSn_2_, Nb_6_Sn_5_ and Nb_3_Sn [15] intermetallics, respectively. The Nb_3_Sn and Nb_3_Al phases are isomorphous, both having the A15 structure (P*m*3*n, cP*8 Cr_3_Si-type). In the Al-Sn phase diagram, there are no intermetallic phases, and Al and Sn show limited solubility for each other [16].

The Al-Nb and Nb-Sn binary phase diagrams contain many phases of interest. Nb-based intermetallics such as NbAl_3_, Nb_2_Al and Nb_3_Al are materials that have promising properties for use at high temperatures [17,18]. These intermetallics have high melting temperatures and low densities, but can exhibit poor oxidation resistance [19]. The electronic properties of the A15 phases, Nb_3_Al and Nb_3_Sn have also been extensively studied for their superconducting applications. Recently, first-principles calculations were used to model the thermodynamic properties, elastic constants and phonon properties of phases in the Nb-Sn and Al-Nb phase diagrams [20,21], whereas high pressure and tensile conditions were modelled for the niobium aluminide phases [22,23].

To the authors’ knowledge, a study of the phase equilibria in the Al-Nb-Sn ternary system has not been reported. There is little information in the literature on the ternary system of Al-Nb-Sn. Bachner et al. [24] found that the two A15 phases, Nb_3_Al and Nb_3_Sn, showed complete solid solubility. Pietzka and Schuster [25] reported the existence of a ternary aluminide phase, Nb_5_Sn_2_Al. They showed its crystal structure was D8_m_ (I4/mcm, tI32 W_5_Si_3_-type) and reported the lattice parameters to be a = b = 1.0629(2) nm, c = 0.5216(2) nm. This phase belongs to a group of ternary compounds with the D8_m_ W_5_Si_3_ prototype structure [26,27,28]. The first such ternary phase reported with this crystal structure was the Nb_5_Sn_2_Si phase [26]. This phase has subsequently been found to be important during the oxidation of Nb-silicide based alloys containing Sn [8,9,10,11], forming at the oxide–substrate interface acting to lower oxygen diffusion. There is little experimental information on this class of materials, and hence any further data will help to understand their potential.

One of the main issues with studying these ternary alloys is that it is difficult to make homogenous alloys. The difference in the melting temperatures of both Nb and Sn is a problem when manufacturing alloys using arc-melting. Extended melting in the arc melter causes the Sn to volatilise, so a compromise between melting to achieve a homogenous alloy and maintaining the composition must be achieved, in particular with high Sn containing alloys. As such, during heat treatments, the alloys require longer times to homogenise and reach equilibrium, especially at 900 °C. Furthermore, it is expected that lower melting temperatures are obtained with increasing amounts of Sn.

The aims of the current work were to verify the stable phases, in particular, the ternary phase, and to establish the phase equilibria in the Nb-rich area of the Al-Nb-Sn phase diagram. In the present study, three alloys that were believed to contain the ternary phase were chosen to define this region of the phase diagram. It was expected that samples with high Al or Sn contents would have a low melting temperature, and as such these regions were avoided.

## 2. Materials and Methods

The actual compositions in at.% of the three Nb-based alloys studied in the current work were Nb-17Al-17Sn (IP4), Nb-33Al-13Sn (IP5) and Nb-16Al-20Sn (IP6). These alloys were studied in as-cast states (AC) and after heat treatments (HT) at 900 °C and 1200 °C. The actual composition was measured using SEM/EDS large area analysis discussed below.

For the preparation of the IP4, IP5 and IP6 alloys, two series of master-alloys instead of pure elements were used. The first contained Nb (99.99 wt.%) and Al (99.99 wt.%) and were in ribbon shape with a nominal composition of Nb-25Al (at.%). The second was produced by melting Sn (99.99 wt.%) with Al (99.99 wt.%) in a tube furnace under Ar-atmosphere to produce an alloy with the composition Al-45Sn (at.%). This was done in order to minimise the evaporation of Sn and Al due to the large difference of melting points of Nb (2477 °C) and Sn (231 °C) or Al (660 °C). The correct proportion of the two master alloys or additional elements were combined to produce the desired compositions.

The alloys were produced by arc-melting under high purity Ar-atmosphere using a non-consumable tungsten electrode and a water-cooled copper crucible. They were re-melted at least three times each to ensure homogeneity. Specimens of cubic geometry were cut for heat treatment and afterwards wrapped in tantalum foil, placed in an alumina boat and annealed in a tube furnace (Lenton Furnaces, Market Harborough, UK under Ti-gettered argon flow. All alloys were annealed at 900 and 1200 °C. The annealing times are given in Table 1. Titanium sponge was used as an oxygen getter and was placed at the entrance of the argon flow in the furnace. All samples were furnace cooled.

The phases in the alloys were identified using X-ray diffraction (XRD)). A Siemens D-5000 diffractometer (Hiltonbrooks Ltd, Crew, UK) with monochromatic Cu Kα (λ = 1.5418 Å) radiation along with JCPDS data were used to identify phases. For Nb_5_Sn_2_Al phase, structural data from the Pearson’s Crystal Data Database were used to calculate the reflection angles. These are given in Appendix A, Table A1. Depending on the Al:Sn ratio in the A15 phase, the XRD peaks corresponded better with Nb_3_Al or Nb_3_Sn diffraction patterns. As these two phases are isomorphous, with complete solid solubility they are hereafter referred to as the A15 phase.

The microstructures of the samples and the chemical analyses of the constituent phases as well as the actual compositions of the alloys were assessed by scanning electron microscopy (SEM), using a JEOL JSM 6400 (JEOL Ltd., Tokyo, Japan) and a FEI Inspect-F scanning electron microscope (ThermoFisher Scientific, Hillsboro, OR, USA). The former instrument was equipped with Oxford Instruments INCA software (Oxford Instruments, High Wycombe, UK) for quantitative chemical analysis, and elemental standards of Nb, Al and Sn. At least ten large area analyses or phase (point) analyses were taken before the average chemical compositions were calculated. A large elemental area scan of the sample was completed to determine the bulk composition of the as-cast alloy. This is the actual composition of the alloys (as opposed to the alloy composition aimed for (nominal)) because of Sn loss during melting.

## 3. Results

### 3.1. Alloy Nb-17Al-17Sn (IP4)

#### 3.1.1. As-Cast

**Figure 1:**
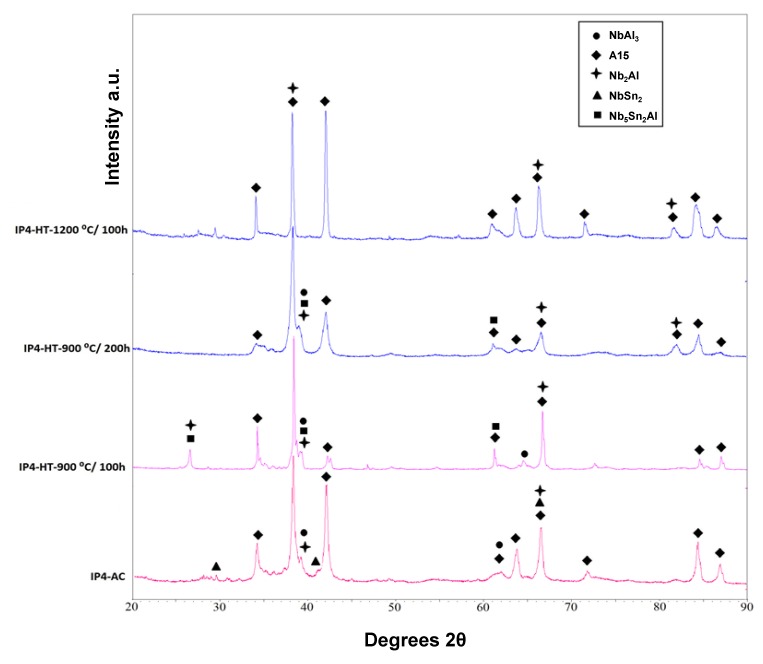
XRD patterns of IP4-AC, IP4-HT-900 °C/100h, IP4-HT-900 °C/200 h and IP4-HT-1200 °C/100 h.

**Figure 2:**
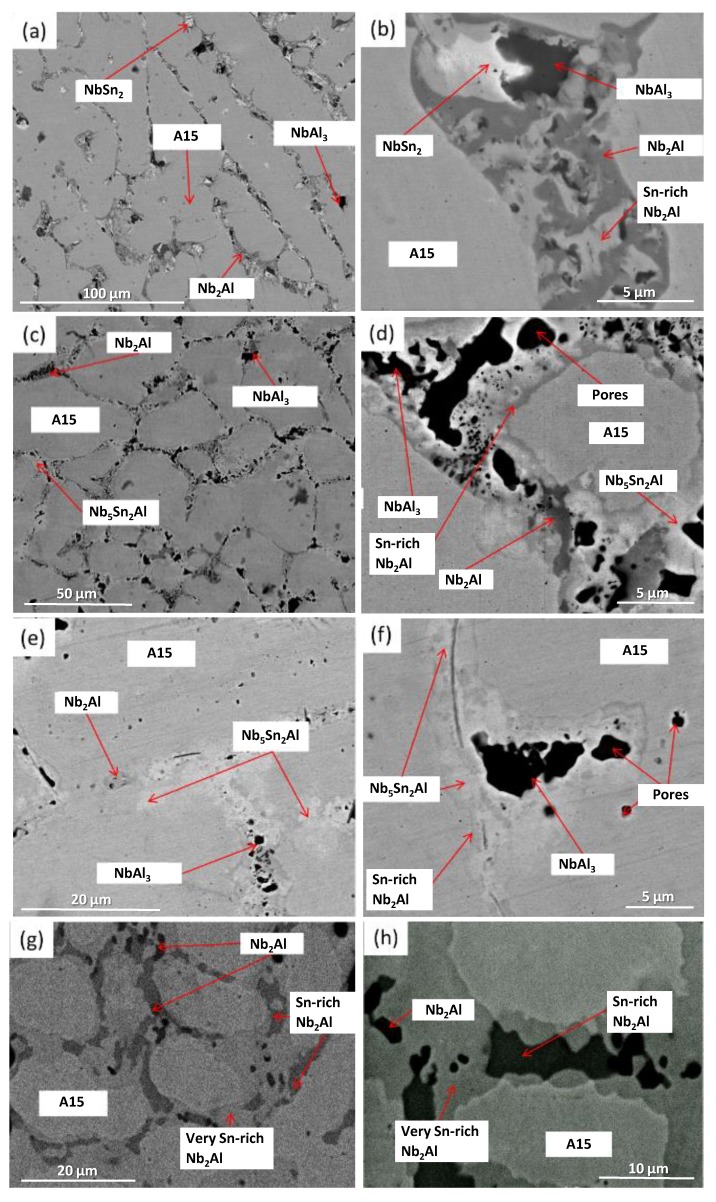
Backscattered electron (BSE) images of (**a**,**b**) IP4-AC, (**c**,**d**) IP4-HT-900 °C/100 h, (**e**,**f**) IP4-HT-900 °C/200 h and (**g**,**h**) IP4-HT-1200 °C/100 h.

XRD showed that A15, NbSn_2_, NbAl_3_ and Nb_2_Al were present (Figure 1). The microstructure of the as-cast alloy (IP4-AC) can be seen in Figure 2a,b and the compositions of the constituent phases are shown in Table 2. Large areas of primary A15 phase were observed, while at the grain boundaries the three other phases were formed: NbSn_2_, NbAl_3_ and Nb_2_Al. The content of Al in A15 was ~10 at.% while in NbSn_2_ was ~7 at.%. The Sn content in NbAl_3_ and Nb_2_Al was nearly 4 at.% and 10 at.%, respectively. Areas of Sn-rich Nb_2_Al were evident (Figure 2b) with a Sn content of ~17 at.%. The Al/Sn ratio and Al + Sn sum in A15 were 0.67 and 25.5 at.%, respectively.

#### 3.1.2. Heat-Treated

**Figure 3:**
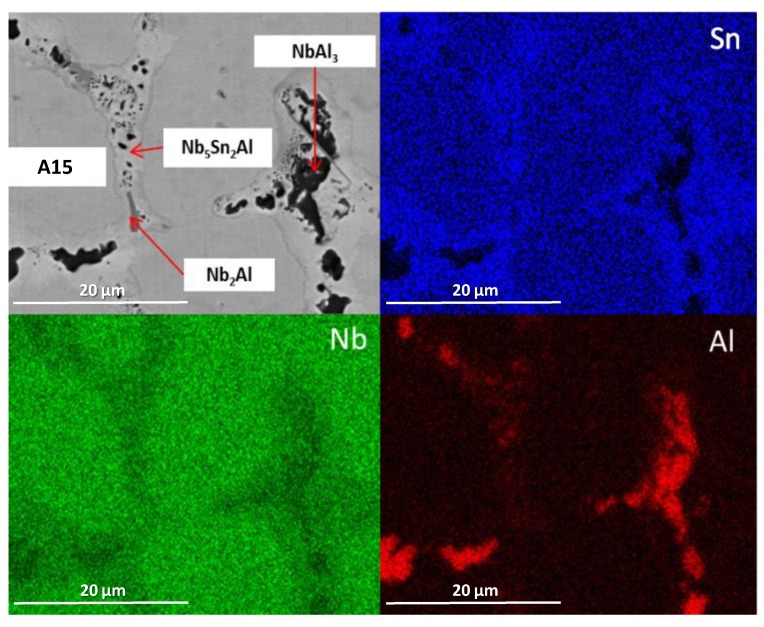
BSE image and X-ray element maps of IP4-HT 900 °C/100 h (for colour, see online version).

The alloy was given three separate heat treatments: 100 h at 900 °C, 200 h at 900 °C and 100 h at 1200 °C. After 100 h at 900 °C (IP4-HT-900 °C/100 h), XRD showed that A15, NbAl_3_, Nb_2_Al and Nb_5_Sn_2_Al were present (Figure 1). NbSn_2_ was not observed. In Figure 2c,d, the microstructure after 100 h at 900 °C is shown. Large areas of A15 phase were evident, while at the grain boundaries NbAl_3_ and Nb_2_Al co-existed with the Nb_5_Sn_2_Al ternary compound. EDS maps of the microstructure of IP4-HT-900 °C/100 h are shown in Figure 3 and composition of phases in Table 2. In the Sn maps, areas with high concentration of Sn were observed, corresponding with the location of the ternary phase. The composition of the ternary phase was Nb-24.9Sn-13.6Al (at.%), which is similar to its stoichiometric formula of Nb_5_Sn_2_Al with slightly higher Al concentration. The Al concentration in the A15 phase was similar to the phase in the as-cast sample. The Al/Sn ratio and Al + Sn sum in A15 was 0.73 and 26.6 at.%, respectively. In Nb_2_Al, the Sn concentration was 9 at.%. Areas of Sn-rich Nb_2_Al were again observed, with the Sn and Al contents being about 17 and 19 at.%, respectively. The NbAl_3_ phase showed a similar composition to the as-cast, but with a lower Sn content (0.7 at.% compared to 3.7 at.%) and was always surrounded by areas containing Nb_5_Sn_2_Al.

IP4 was heat treated for an additional 100 h at 900 °C to see if equilibrium could be achieved. The same constituent phases were present in the alloy IP4-HT-900 °C/200 h (Figure 1 and Figure 2e,f)). No significant changes occurred in the compositions of the phases (Table 2). The Al/Sn ratio (0.75) and Al + Sn sum (26.7 at.%) for A15 phase did not change. However, some features of the phases at the grain boundaries were altered. At the grain boundaries, only remnants of NbAl_3_ were evident. Pores were observed where NbAl_3_ previously existed (Figure 2f). Comparing XRD patterns, it appears that NbAl_3_ peaks are less intense after heat treatment, while those attributed to A15 and Nb_2_Al are more intense (Figure 1).

IP4 was also heat treated at 1200 °C for 100 h. XRD showed two phases: Nb_2_Al and A15 (Figure 1). The microstructure is shown in Figure 2g,h. Areas of Sn-rich and very Sn-rich Nb_2_Al phase were evident throughout the alloy. Tin content varied between ~2–6 and ~18 at.%. The Al content in A15 was 9.6 at.%, which was slightly less than in the as-cast and the heat-treated alloys at 900 °C. The ratio of Al/Sn was 0.62 and the Al + Sn sum 25.0 at.% in A15, which was again slightly less than measured in the as-cast and 900 °C heat-treated alloys.

### 3.2. Alloy Nb-33Al-13Sn (IP5)

#### 3.2.1. As-Cast

**Figure 4:**
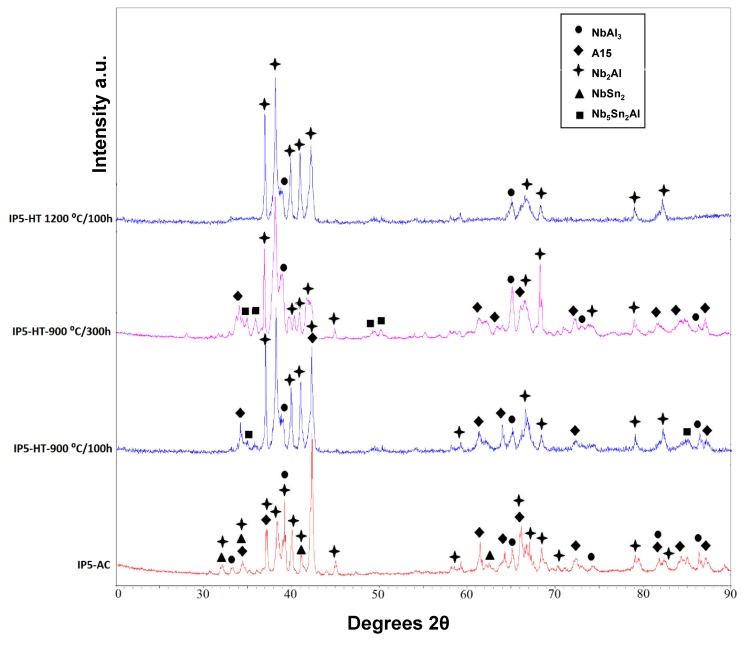
XRD patterns of IP5-AC, IP5-HT-900 °C/100 h, IP5-HT-900 °C/300 h and IP5-HT-1200 °C/100 h**.**

**Figure 5:**
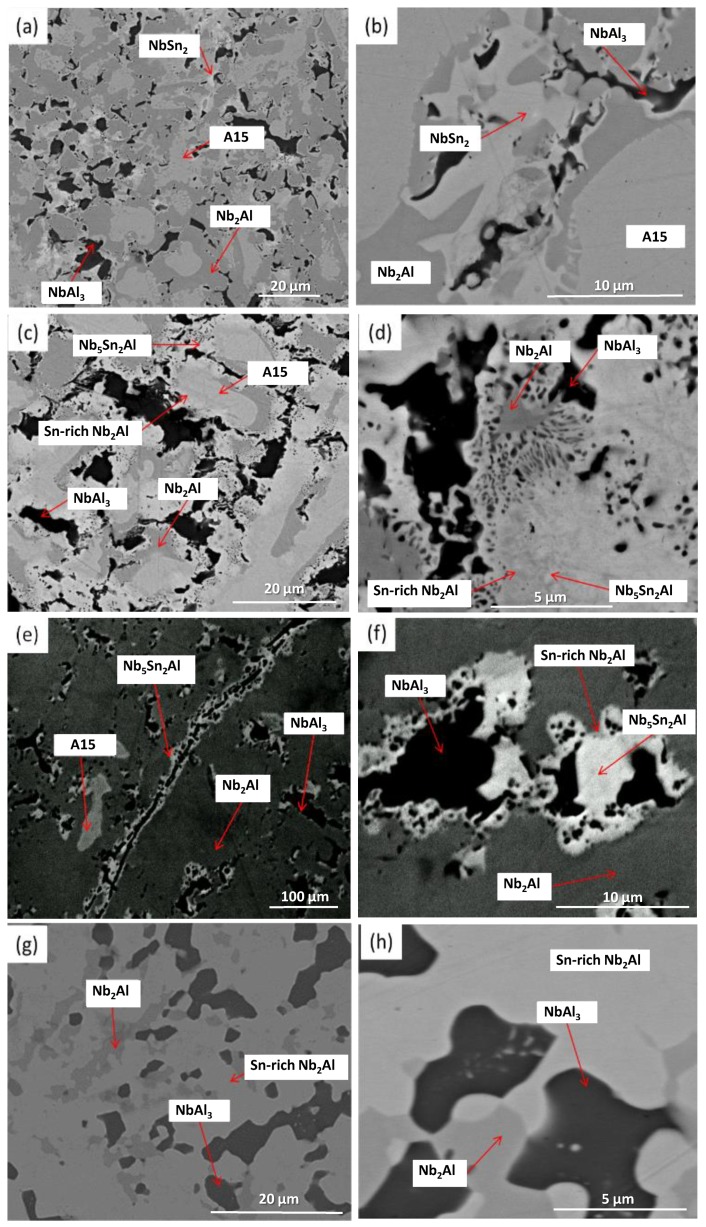
BSE images of (**a**,**b**) IP5-AC, (**c**,**d**) IP5-HT-900 °C/100 h, (**e**,**f**) IP5-HT-900 °C/300 h and (**g**,**h**) IP5-HT-1200 °C/100 h.

XRD showed that four phases were present in the as-cast alloy (IP5-AC): A15, NbAl_3_, NbSn_2_ and Nb_2_Al (Figure 4). The microstructure and the compositions of the phases present in IP5-AC can be seen in Figure 5a,b and Table 3, respectively. The SEM images show that A15 has the largest volume fraction. In the A15 phase, the Sn content was ~12 at.%, while in Nb_2_Al it was ~7 at.%. Of all the phases, Sn showed the least solubility in NbAl_3_ of less than 2 at.%. The ratio Al/Sn in the A15 phase was 1.41 while the sum of Al + Sn was 29.2 at.%.

#### 3.2.2. Heat-Treated

**Figure 6:**
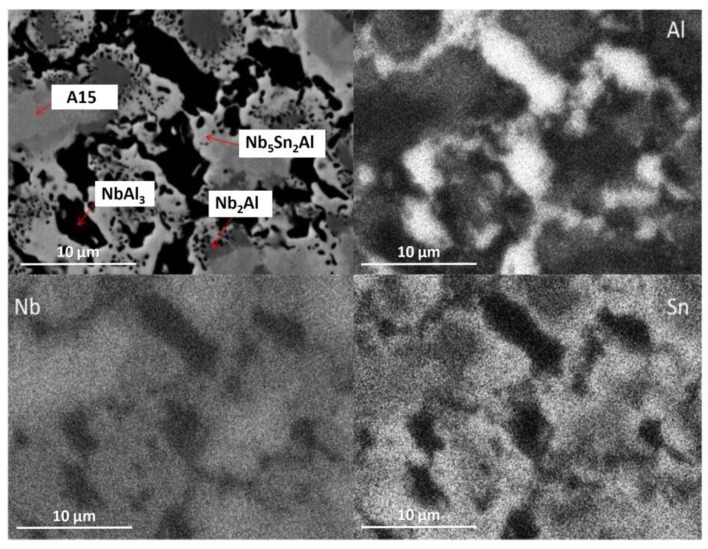
BSE image and X-ray element maps of IP5-HT 900 °C/100 h.

IP5-AC was given three separate heat treatments: 100 h at 900 °C, 300 h at 900 °C and 100 h at 1200 °C. After 100 h at 900 °C (IP5-HT-900 °C/100 h), the phases A15, Nb_5_Sn_2_Al, NbAl_3_ and Nb_2_Al were observed (Figure 4). The NbSn_2_ intermetallic was again no longer present. In Figure 5c,d, the microstructure of the alloy after heat treatment at 900 °C for 100 h can be seen. Large areas of A15 were again evident as in the as-cast alloy, with the Al/Sn ratio decreasing to 1.33. The Al + Sn sum was similar at 28.7 at.%. The Nb_5_Sn_2_Al ternary compound was formed along with NbAl_3_ and Nb_2_Al. EDS maps show areas with high Sn concentrations, which correspond with the location of the ternary phase (Figure 6). The composition of Nb_5_Sn_2_Al was close to stoichiometry (Nb-13.2 Al-24.9 Sn (at.%)) again, slightly enriched in Al. The concentration of Sn in A15, Nb_2_Al and NbAl_3_ was similar to the as-cast, except that the Sn content of NbAl_3_ was slightly lower at 0.7 at.%. Aluminium contents were also similar, except for in Nb_2_Al where the Al concentration decreased by ~3.5 at.%. Some areas of Sn-rich Nb_2_Al were again observed, with the Sn and Al content being more than 20 and 19 at.%, respectively.

IP5 was heat treated for a further 200 h at 900 °C (IP5-HT-900 °C/300 h) to achieve an equilibrium microstructure. Although a significant change in the microstructure was seen (volume fraction of phases), no major change in compositions was observed (Figure 5e,f, Table 3). Nb_2_Al became the dominant phase, whereas only traces of the A15 phase were found (Figure 5e). XRD showed less intense peaks for A15 (Figure 4). At the grain boundaries NbAl_3_, Nb_5_Sn_2_Al and Sn-rich Nb_2_Al were present (Figure 5f).

The IP5-AC alloy was heat treated at 1200 °C for 100 h. Two phases were identified by XRD and SEM/EDS: NbAl_3_ and Nb_2_Al (Figure 4 and Figure 5g,h). Neither NbSn_2_ nor the ternary phase, Nb_5_Sn_2_Al, was observed. Areas of Sn-rich Nb_2_Al were evident throughout the alloy, with a larger volume fraction than the Nb_2_Al with a lower Sn content (Figure 5g,h). Compared with the as-cast sample, the NbAl_3_ intermetallic had approximately the same composition, while Nb_2_Al was poorer in Sn by nearly 3 at.% (Table 3). The Sn content in the Sn-rich Nb_2_Al phase decreased compared with the corresponding phase in the 900 °C heat-treated alloy.

### 3.3. Alloy Nb-16Al-20Sn (IP6)

#### 3.3.1. As-Cast

**Figure 7:**
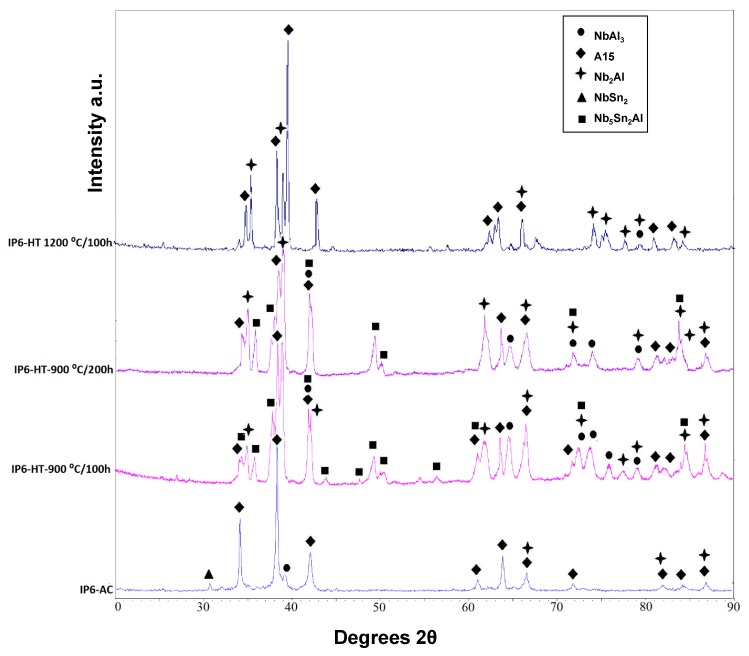
XRD patterns of IP6-AC, IP6-HT-900 °C/100 h, IP6-HT-900 °C/200 h and IP6-HT-1200 °C/100 h.

**Figure 8:**
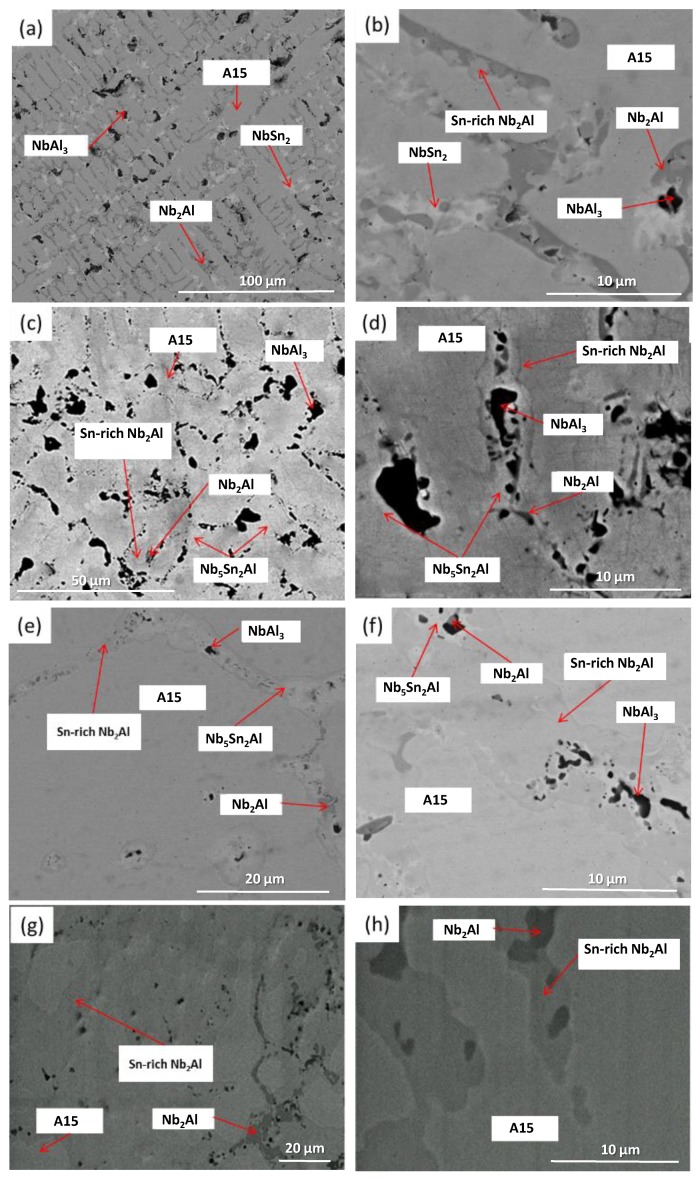
BSE images of (**a**,**b**) IP6-AC, (**c**,**d**) IP6-HT-900 °C/100 h, (**e**,**f**) IP6-HT-900 °C/200 h and (**g**,**h**) IP6-HT-1200 °C/100 h.

XRD of the as-cast alloy (IP6-AC) shows the phases A15, Nb_2_Al, NbSn_2_ and NbAl_3_ (Figure 7). In Figure 8a,b, BSE images of the IP6-AC are shown. The compositions of phases A15, Nb_2_Al, NbSn_2_ and NbAl_3_ are given in Table 4. The dominant phase was A15, which contained more Sn than Al. The other phases were accumulated at the grain boundaries. In these areas, Sn-rich Nb_2_Al was also observed (Figure 8b). The concentration of Al in A15 was ~9 at.%, while the Al/Sn ratio was 0.50 and the Al + Sn sum 25.9 at.%. The NbSn_2_ phase contained ~6.8 at.% Al. NbAl_3_, Nb_2_Al and the Sn-rich Nb_2_Al contained 1.6, 7.7 and 17.2 at.% Sn, respectively.

#### 3.3.2. Heat-Treated

**Figure 9:**
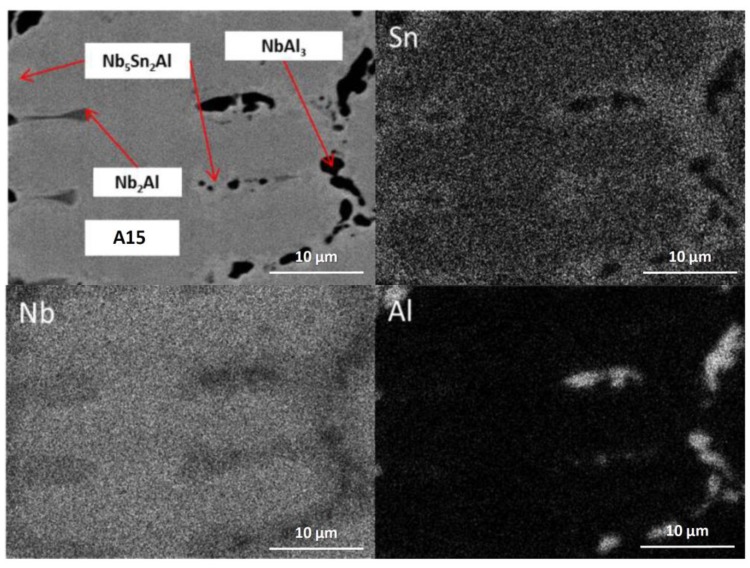
BSE image and X-ray element maps of IP6-HT 900 °C/100 h.

The alloy was given three separate heat treatments: 100 h at 900 °C, 200 h at 900 °C and 100 h at 1200 °C. After 100 h at 900 °C (IP6-HT-900 °C/100 h), A15, Nb_2_Al and NbAl_3_ were still present along with newly formed Nb_5_Sn_2_Al (Figure 7 and Figure 8c,d), with its composition being close to stoichiometry (Table 4). The bright areas in the X-Ray element map of Sn in Figure 9 suggest the area where the ternary compound is present. NbSn_2_ was no longer observed. A15 was again the matrix phase, while the other phases were present at the phase boundaries (Figure 8c,d).

The compositions of the phases are shown in Table 4. The composition of A15 was similar to the as-cast, with the Al/Sn ratio increasing slightly to 0.55 and the Al + Sn sum less than 27 at.%. The Sn concentration in Nb_2_Al decreased, whereas in Sn-rich Nb_2_Al it increased, in both cases by about 2 at.%. The NbAl_3_ intermetallic had a similar composition to the phase in the as-cast alloy.

The XRD data (Figure 7) along with the EDS analysis (Table 4) confirmed that after a further 100 h at 900 °C (IP6-HT-900 °C/200 h), the composition of the constituent phases did not undergo any notable changes compared with IP6-HT-900 °C/100 h. The same does not apply to the microstructure as a significant alteration took place. The volume fraction of NbAl_3_ decreased considerably (Figure 8e,f). A15 continued to be the matrix phase, whereas the rest of the phases again accumulated at the grain boundaries.

The IP6-AC alloy was annealed at 1200 °C for 100 h (IP6-HT-1200 °C/100 h). XRD showed only the presence of A15 and Nb_2_Al (Figure 7). The microstructure was significantly altered after this heat treatment compared with the as-cast. In the heat-treated sample, only A15 (matrix) and Nb_2_Al were observed. The NbSn_2_ and NbAl_3_ were no longer present, while the dark areas were pores (Figure 8g,h). Nb_2_Al again formed Sn-rich areas with larger volume fraction than the Nb_2_Al with low Sn content. As it can be seen in Table 4, the composition of A15 was approximately the same compared with the IP6-AC, with the Al/Sn ratio being 0.49 and Al + Sn sum being 26.0 at.%. Sn content in Nb_2_Al decreased by ~3 to 4.6 at.% compared with as-cast, while in Sn-rich Nb_2_Al the concentration of Sn increased by ~4 to ~21 at.%.

## 4. Results and Discussion

All the as-cast alloys in the present study contained NbSn_2_, NbAl_3_, Nb_2_Al and A15. The NbSn_2_ phase was metastable, disappearing after heat treatment at 900 °C and 1200 °C. For IP4 and IP6 after heat treatment at 900 °C for 200 h, the fraction of NbAl_3_ diminished significantly, thus it is concluded that this phase was also metastable at the given compositions. For the IP5 alloy, only remnants of A15 phase were present after 300 h of annealing at 900 °C, indicating that for this composition it was not an equilibrium phase. In NbAl_3_ and Nb_2_Al, the solubility of Sn was found to be up to 3.7 (IP4) and 9.8 at.% (IP4), respectively. In IP4 and IP6, Sn-rich areas of Nb_2_Al were observed with Sn solubility up to about 18 at.% (IP4), whereas in IP5 no such areas were seen. In both IP4 and IP6, the Al/Sn ratio in A15 increased after heat treatment at 900 °C, whereas it remained constant after annealing at 1200 °C compared to the as-cast alloys. In IP5, the Al/Sn ratio decreased slightly in A15 after annealing at 900 °C. The Al + Sn sum in A15 phases in IP4 and IP6 was ~25–27 at.%, while in IP5 it was ~29 at.%.

It is clear that the Nb_5_Sn_2_Al compound was present in all the heat-treated specimens at 900 °C. It primarily formed at the grain boundaries, co-existing in most cases with NbAl_3_ and Nb_2_Al. The ternary compound was, however, not present at 1200 °C. This suggests that the phase is stable up to a temperature between 900 and 1200 °C. Its stability appears to be similar to the ternary phase Nb_5_Sn_2_Si, which is reported to be stable up to 1200 °C [8,10,29]. Its composition was found to be close to stoichiometry, similar to the Nb_5_Sn_2_Si phase.

From the microscopy and XRD, it is clear that the samples at 900 °C have not reached equilibrium even after 300 h heat treatment, since more than 3 phases are observed. This is due to the method of preparation, and the difficulty of homogenising these alloys during manufacturing in the arc melter without losing significant amounts of Sn. However, analysing the samples at 100, 200 and 300 h shows a clear progression towards equilibrium-phase assemblage. In IP4 and IP6, the amount of NbAl_3_ diminished with increasing heat-treatment time. In IP5, the A15 phases deceased significantly when the heat treatment time was extended, and only remnants remained after 300 h. This indicates that these two phases are metastable at the given compositions. This strongly suggests that the equilibrium phase regions for IP4 and IP6 are A15 (Nb_3_ (Al, Sn)) + Nb_5_Sn_2_Al + Nb_2_Al at 900 °C and A15 (Nb_3_ (Al, Sn)) + Nb_2_Al at 1200 °C. For IP5, the equilibrium phase regions are most likely NbAl_3_ + Nb_2_Al + Nb_5_Sn_2_Al at 900 °C and NbAl_3_ + Nb_2_Al at 1200 °C.

At 1200 °C, it is expected that a wide two phases region between A15 and Nb_2_Al exists, since there was no evidence of melting of the alloys IP4 and IP6 during heat treatment at 1200 °C. This suggests that the solubility of Sn in Nb_2_Al is positioned towards the composition of the measured Sn-rich Nb_2_Al phases. Based on the present work, the ternary phase would not be stable at 1200 °C.

Microscopy of the as-cast samples showed that the A15 phase is the primary phase in the three alloys. In IP4 and IP6, A15 was richer in Sn than Al, whereas in IP5 the A15 phase was richer in Al than Sn. In IP4 and IP6, as the A15 phase formed first in the melt, the latter became leaner in Sn but richer in Al near the A15 phase. Thus Nb_2_Al was formed, along with Sn-rich areas between the Nb_2_Al and A15. After that and as the melt was still rich in Al, the NbAl_3_ phase was formed. The last phase to solidify was NbSn_2_, as the melt became leaner in Al and richer in Sn. For IP5, a similar solidification path was observed. A15 formed first from the melt. The removal of Sn allowed the formation of Nb_2_Al followed by NbAl_3_. Again NbSn_2_ was the last phase to form as the melt became Sn rich. It is suggested that the solidification path for the three alloys was L→L + A15→L + A15 + Nb_2_Al→ L + A15 + Nb_2_Al + NbAl_3_→A15 + Nb_2_Al + NbAl_3_ + NbSn_2_.

## 5. Conclusions

Phase equilibria in the Nb-Al-Sn phase diagram at 900 and 1200 °C are reported. Despite some of the alloys not reaching equilibrium after heat treatment at 900 °C, an equilibrium phase assemblage could be inferred. The stable phases in Nb-17Al-17Sn (IP4) and Nb-16Al-20Sn (IP6) at 900 °C were A15, Nb_5_Sn_2_Al and Nb_2_Al, whereas at 1200 °C the phases were A15 and Nb_2_Al. For Nb-33Al-13Sn (IP5) at 900 °C, the equilibrium phases were Nb_2_Al, Nb_5_Sn_2_Al and NbAl_3_, and at 1200 °C were Nb_2_Al and NbAl_3_. The Nb_2_Al shows a high solubility for Sn, reaching 21 at.% in the Sn-rich areas. The A15 phase showed complete solubility between the two end members Nb_3_Al and Nb_3_Sn. The solubility of Sn in NbAl_3_ was limited, and did not exceed 3.7 at.%. In the metastable phase NbSn_2_, the solubility of Al varied between 4.8 and 6.8 at.%. The Nb_5_Sn_2_Al ternary intermetallic phase was observed in all samples, forming at the grain boundaries after heat treatment at 900 °C. The phase was near stoichiometric and was stable at 900 °C, but not seen after heat treatment at 1200 °C. The phase equilibria data will be useful for further alloy development of Nb-silicide based alloys and coatings containing Al and Sn. Improving the oxidation resistance of these materials will enable their application in aero engines at high operating temperatures.

## Figures and Tables

**Table 1:** Annealing times for samples at 900 and 1200 °C.

Sample	Annealing Temperature	
	900 °C	1200 °C
IP4 (Nb-17Al-17Sn)	100, 200 h	100 h
IP5 (Nb-33Al-13Sn)	100, 300 h	100 h
IP6 (Nb-16Al-20Sn)	100, 200 h	100 h

**Table 2:** EDS analysis of the phases in IP4-AC, IP4-HT 900 °C/100 h, IP4-HT 900 °C/200 h and IP4-HT 1200 °C/100 h alloys.

Sample	Phases	Nb (at.%)	Al (at.%)	Sn (at.%)
IP4-AC	A15	74.5 ± 0.6	10.2 ± 0.6	15.3 ± 0.3
	NbAl_3_	25.9 ± 0.4	70.4 ± 0.3	3.7 ± 0.2
	Nb_2_Al	59.5 ± 0.3	30.7 ± 0.3	9.8 ± 0.4
	NbSn_2_	34.9 ± 0.3	6.5 ± 0.5	58.6 ± 0.5
	Sn-rich Nb_2_Al	64.9 ± 0.2	18.0 ± 0.3	17.1 ±0.3
IP4-HT 900 °C /100 h	A15	73.4 ± 0.5	11.2 ± 0.4	15.4 ± 0.3
	Nb_5_Sn_2_Al	61.5 ± 0.4	13.6 ± 0.5	24.9 ± 0.3
	NbAl_3_	27.0 ± 0.2	72.3 ± 0.4	0.7 ± 0.1
	Nb_2_Al	65.9 ± 0.2	25.1 ± 0.3	9.0 ± 0.2
	Sn-rich Nb_2_Al	63.3 ± 0.5	19.4 ± 0.3	17.3 ± 0.3
IP4-HT 900 °C /200 h	A15	73.3 ± 0.5	11.4 ± 0.4	15.3 ± 0.2
	Nb_5_Sn_2_Al	62.5 ± 0.5	13.0 ± 0.1	24.5 ± 0.3
	NbAl_3_	26.4 ± 0.2	72.2 ± 0.3	1.4 ± 0.1
	Nb_2_Al	65.8 ± 0.6	25.4 ± 0.2	8.8 ± 0.1
	Sn-rich Nb_2_Al	62.7 ± 0.6	20.3 ± 0.3	17.0 ± 0.2
IP4-HT 1200 °C /100 h	A15	75.0 ± 0.6	9.6 ± 0.1	15.4 ± 0.2
	Nb_2_Al	62.4 ± 0.6	35.6 ± 0.3	2.0 ± 0.1
	Sn-rich Nb_2_Al	65.1 ± 0.5	28.8 ± 0.3	6.1 ± 0.2
	Very Sn-rich Nb_2_Al	63.1 ± 0.2	19.1 ± 0.1	17.8 ± 0.1

**Table 3:** EDS analysis of the phases in IP5-AC, IP5-HT 900 °C/100 h, IP5-HT 900 °C/300 h and IP5-HT 1200 °C/100 h alloys.

Sample	Phase	Nb (at.%)	Al (at.%)	Sn (at.%)
IP5-AC	A15	70.8 ± 0.6	17.1 ± 0.4	12.1 ± 0.3
	NbAl_3_	26.3 ± 0.4	72.0 ± 0.6	1.7 ± 0.1
	Nb_2_Al	58.2 ± 0.4	34.4 ± 0.3	7.4 ± 0.2
	NbSn_2_	31.5 ± 1.5	4.8 ± 0.4	63.7 ± 1.4
IP5-HT-900 °C /100 h	A15	71.3 ± 0.6	16.4 ± 0.4	12.3 ± 0.2
	Nb_5_Sn_2_Al	61.9 ± 0.4	13.2 ± 0.1	24.9 ± 0.4
	NbAl_3_	26.9 ± 0.4	72.4 ± 0.6	0.7 ± 0.1
	Nb_2_Al	62.3 ± 0.6	30.9 ± 0.2	6.8 ± 0.1
	Sn-rich Nb_2_Al	60.2 ± 0.6	19.5 ± 0.2	20.3 ± 0.4
IP5-HT-900 °C /300 h	A15	71.4 ± 0.5	16.7 ± 0.4	11.9 ± 0.2
	Nb_5_Sn_2_Al	62.7 ± 0.4	13.0 ± 0.3	24.3 ± 0.5
	NbAl_3_	26.6 ± 0.2	73.1 ± 0.4	0.3 ± 0.1
	Nb_2_Al	61.1 ± 0.3	33.6 ± 0.2	5.3 ± 0.1
	Sn-rich Nb_2_Al	61.2 ± 0.5	18.1 ± 0.2	20.7 ± 0.2
IP5-HT-1200 °C /100 h	A15	26.9 ± 0.4	72.4 ± 0.4	0.7 ± 0.1
	Nb_2_Al	62.9 ± 0.4	32.4 ± 0.5	4.7 ± 0.1
	Sn-rich Nb_2_Al	62.6 ± 0.5	20.1 ± 0.4	17.3 ± 0.3

**Table 4:** EDS analysis of the phases in IP6-AC, IP6-HT 900 °C/100 h, IP6-HT 900 °C/200 h and IP6-HT 1200 °C/100 h alloys.

Sample	Phase	Nb (at.%)	Al (at.%)	Sn (at.%)
IP6-AC	A15	74.1 ± 0.5	8.6 ± 0.3	17.3 ± 0.3
	NbAl_3_	27.0 ± 0.4	71.4 ± 0.5	1.6 ± 0.2
	Nb_2_Al	60.2 ± 0.6	32.1 ± 0.4	7.7 ± 0.2
	NbSn_2_	30.2 ± 0.9	6.8 ± 0.2	63.0 ± 1.3
	Sn-rich Nb_2_Al	65.7 ± 0.6	17.1 ± 0.3	17.2 ± 0.3
IP6-HT-900 °C/100 h	A15	73.1 ± 0.6	9.6 ± 0.3	17.3 ± 0.3
	Nb_5_Sn_2_Al	62.5 ± 0.6	11.9 ± 0.3	25.6 ± 0.4
	NbAl_3_	27.8 ± 0.5	71.0 ± 0.5	1.2 ± 0.1
	Nb_2_Al	60.6 ± 0.4	34.1 ± 0.4	5.3 ± 0.2
	Sn-rich Nb_2_Al	64.3 ± 0.5	16.2 ± 0.3	19.5 ± 0.2
IP6-HT-900 °C/200 h	A15	73.3 ± 0.4	9.4 ± 0.2	17.3 ± 0.3
	Nb_5_Sn_2_Al	62.8 ± 0.4	12.3 ± 0.2	24.9 ± 0.3
	NbAl_3_	26.8 ± 0.3	72.3 ± 0.3	0.9 ± 0.1
	Nb_2_Al	61.7 ± 0.5	32.9 ± 0.4	5.4 ± 0.1
	Sn-rich Nb_2_Al	64.2 ± 0.5	17.1 ± 0.3	18.7 ± 0.2
IP6-HT-1200 °C/100 h	A15	74.0 ± 0.6	8.5 ± 0.3	17.5 ± 0.3
	Nb_2_Al	60.3 ± 0.4	35.1 ± 0.3	4.6 ± 0.2
	Sn-rich Nb_2_Al	62.5 ± 0.4	16.2 ± 0.3	21.3 ± 0.3

## Appendix A

**Table A1:** Calculated peak positions for Nb_5_Sn_2_Al based on the lattice constants provide in [25]. Where h, k, l refer to the Miller indices, d corresponds to the d-spacing and θ, 2θ refer to the diffraction angle.

h	k	l	d	θ	2Θ
0	0	2	0.2608	17.178	34.356
1	1	0	0.751584	5.882204	11.76441
1	1	2	0.246388	18.21701	36.43402
2	0	0	0.53145	8.333438	16.66688
2	0	2	0.234128	19.20728	38.41456
2	2	2	0.214258	21.0693	42.1386
2	1	1	0.351336	12.66408	25.32817
2	2	0	0.375792	11.82758	23.65515
2	1	3	0.163287	28.1458	56.29159
3	2	1	0.256643	17.46515	34.93029
3	3	0	0.250528	17.90567	35.81134
3	1	0	0.336118	13.24764	26.49528
3	1	2	0.206049	21.95136	43.90272
3	3	2	0.180672	25.23452	50.46903
3	2	3	0.14976	30.95222	61.90445
4	0	0	0.265725	16.85001	33.70003
4	2	0	0.237672	18.90994	37.81988
4	1	1	0.231106	19.46849	38.93698
4	4	0	0.187896	24.20071	48.40142
4	0	2	0.18613	24.44522	48.89044
4	1	3	0.144146	32.3001	64.60021
5	2	1	0.184601	24.66116	49.32231
5	3	0	0.182286	24.99572	49.99145
6	0	0	0.17715	25.77263	51.54527
6	2	0	0.168059	27.27879	54.55757
6	3	1	0.151607	30.53447	61.06894
7	1	0	0.150317	30.82502	61.65004
4	2	4	0.114323	42.3569	84.71381
7	1	2	0.130233	36.2591	72.5182
